# Supplementary material for: Coordination of LMO7 with FAK Signaling Sustains Epithelial Integrity in Renal Epithelia Exposed to Osmotic Pressure
Source: Cells. 2022 Nov 28;11(23):3805. doi: 10.3390/cells11233805 (PMC9741450; doi:10.3390/cells11233805)
Supplement: Supplementary file 1 [file cells-11-03805-s001.zip › cells-1901045-supplementary.pdf]

Supplementary method

Cell Culture, secretome preparation, and secretome profiling

NERK52E cells were purchased from ATCC. The 10^5 cells were seeded on 100 mm peri dish in DMEM medium supplemented with 10% fetal bovine serum (FBS) at 37 °C in a humidified atmosphere at 5% CO_2 for two days. Then, those DMEM media were removed. For secretome profiling, cells were cultured in 10 mL serum free isotonic DMEM, and 10 mL 620 mOsm/kg hypertonic DMEM for 24 hours, respectively. Then, the conditioned media were collected. The conditioned media were concentrated with with Spin-X Concentrators (Life Sciences, Tewksbury, MA, USA).

To profile secretome, the secretome proteins 10 g was subjected to proteomic analysis. The Ingenuity pathway analysis (IPA) was applied to analyze proteins in isotonic srectome and hypertonic secretome. Prediction of IPA analysis and Genecards was described in table S1, supplementary table S2, and supplementary figure S1 A,B.

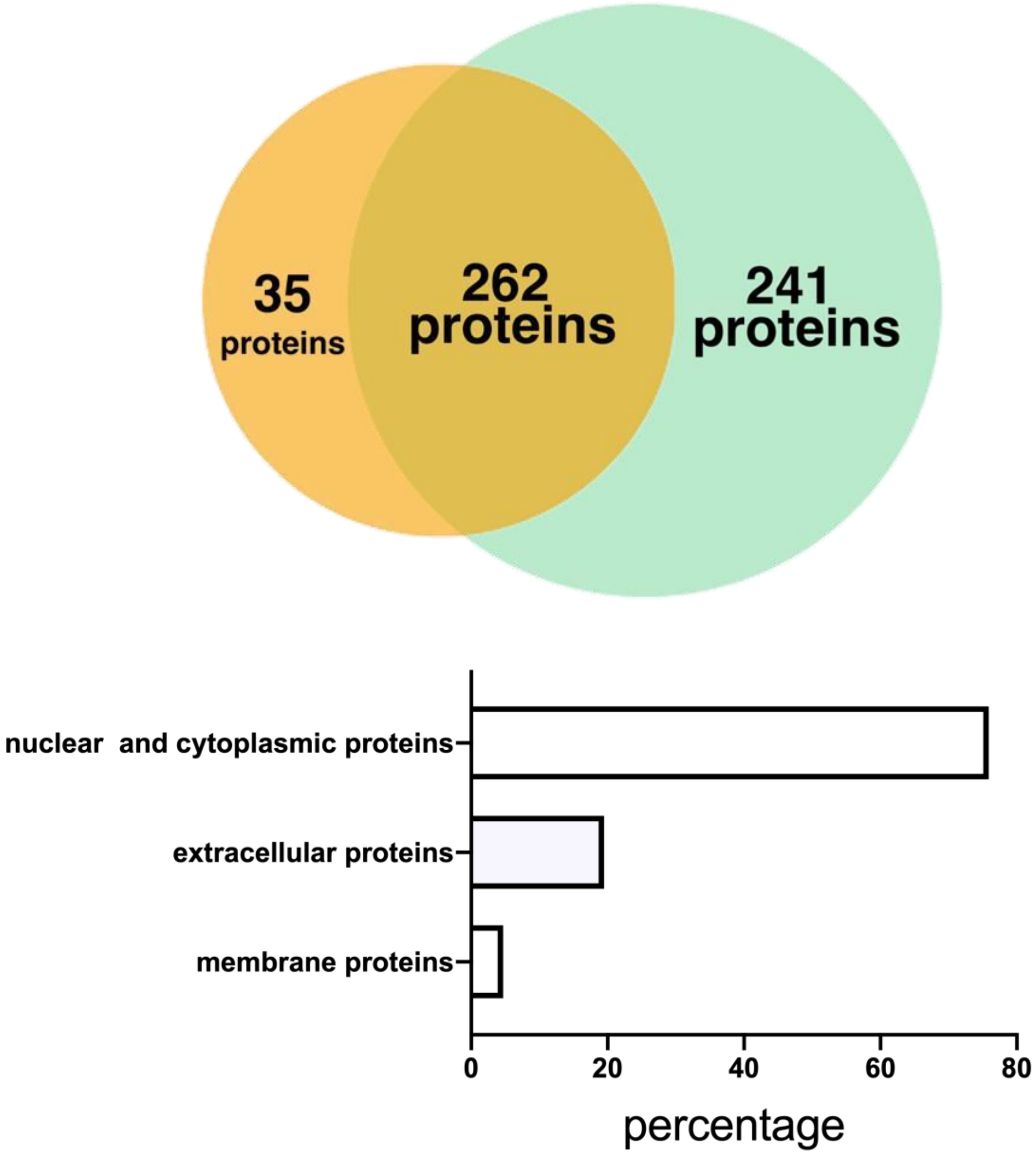

Figure S1. Result of pathway analysis for proteins identified in hypertonic secretome.

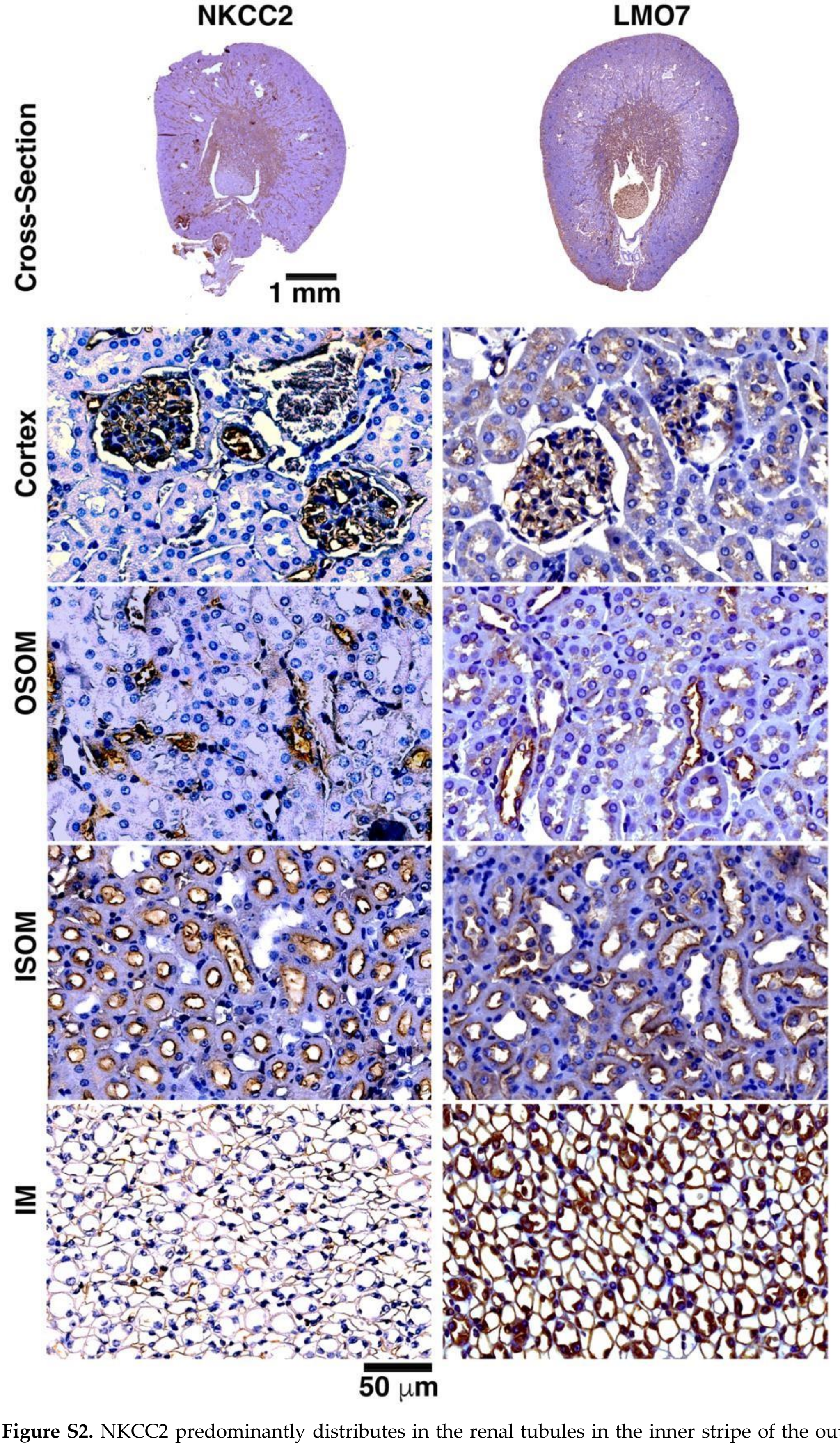

Figure S2. NKCC2 predominantly distributes in the renal tubules in the inner stripe of the outer medulla (OSOM) and inner medulla (IM). The kidney biopsies were probed with antibodies against NKCC2 and LMO7. Immunohistochemical staining showed that the NKCC2 was detected in glo-

meruli, renal tubules in ISOM, and IM. Similarly, the LMO7 also expressed in glomeruli, renal tubules in ISOM, and IM. LMO7 and NKCC2 were part of renal tubules in the outer stripe of the outer medulla (OSOM).

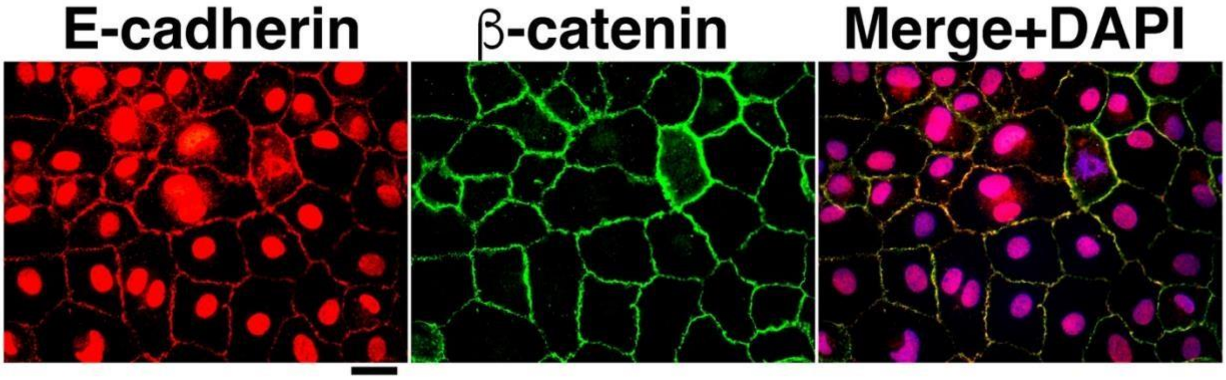

Figure S3. NRK-52E epithelial sheet bears epithelial character. NRK-52E cells were cultured in DMEM supplemented 10% FBS for 4 days to grow epithelial sheet. The epithelial character of NRK-52E epithelial sheet was characterized with immunofluoresent staining, which implied the E-cadherin (red) and β-catenin (green) both are present in cell-cell junction ins the boundary between two adjacent cells. The E-cadherin and β-catenin both are present in junctional area, and loss of junctional integrity is absent in the NEK52E epithelial sheet. Bar = 20 μm.

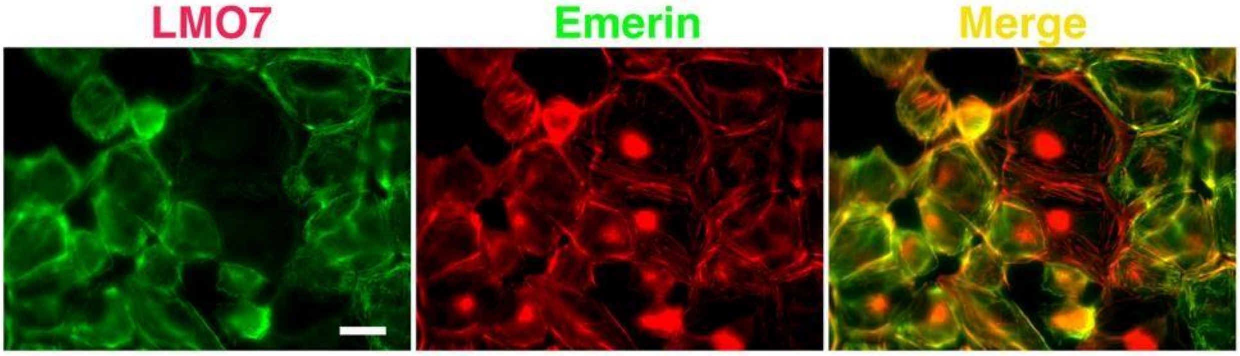

Figure S4. Disordered F-actin fibers are present in cortex of NRK-52E cells with LMO7 depletion. The NRK-52E cells were transfected with shRNA targeted to LMO7. Antibody against LMO7 (green) and Alexa-584 conjugated phalloidin (red) were subjected to stain LMO7 and F-actin. The cortical F-actin stress fibers are not disorderedly distributed in junctional boundaries in the cells with LMO7 depletion. Bar = 20 μm.

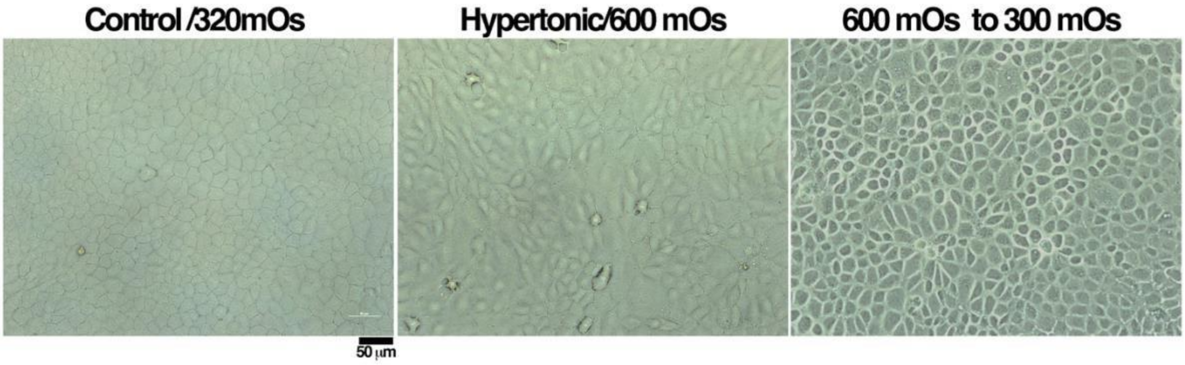

Figure S5. Osmotic alternation leads to broaden the junctional linkage between two adjacent cells. The NRK-52E epithelial sheets were cultured in 320 mOsm/kg isotonic, 620 mOsm/kg hypertonic medium, and 620/320 mOsm/kg osmotic alternation (including 620mOsm/kg an hour and following 320 mOsm/kg for another hour) for two hours, respectively. Epithelial sheets were visualized with Nomarski interference contrast. The interfaces between two adjacent cells are getting bigger in the cells cultured in 620/320 mOsm/kg hypertonic-isotonic alternation.

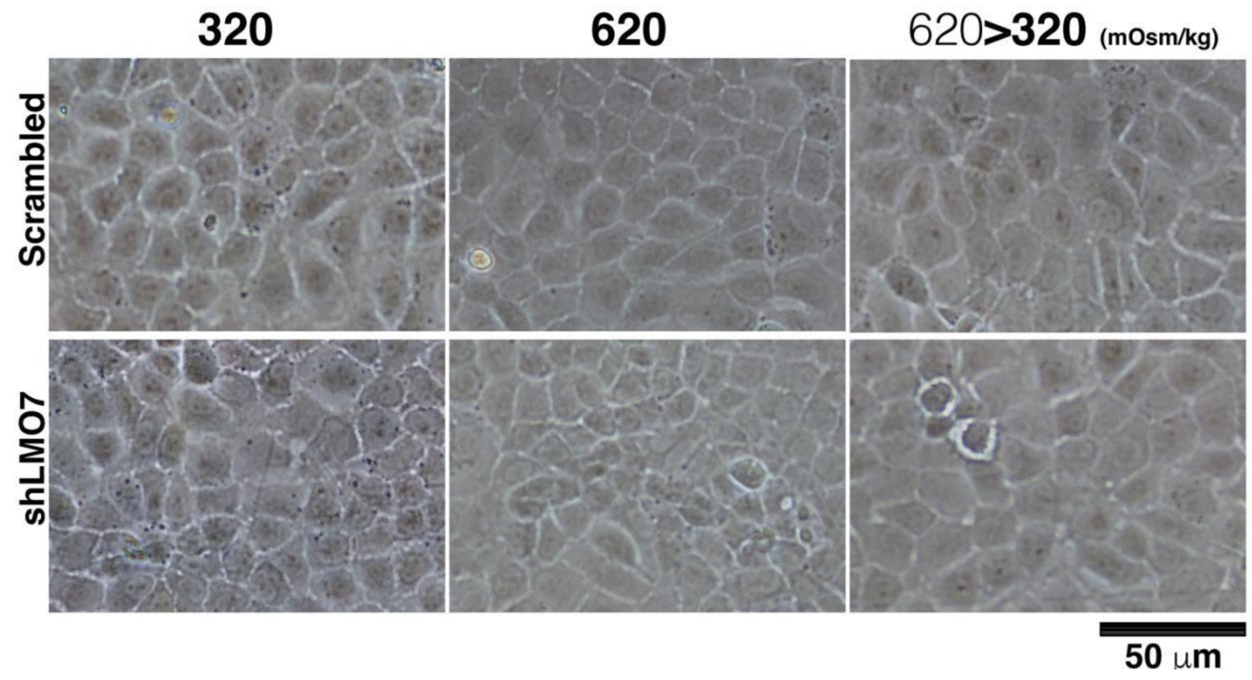

Figure S6. Loss of junctional integrity is apparently present in the shLMO7 depleted NRK52E epithelial sheet. The epithelial sheets were visualized with Nomarski interference contrast microscopy. Under isotonic-hypertonic alternation, although epithelia in epithelial sheets displayed boundaries between two adjacent cells were broadening, loss of junctional integrity is not visible in the NRK-52E epithelial sheet without LMO7 depletion. Boundaries between two adjacent cells are widening in the LMO7 depleted NRK-52E epithelial sheet under hypertonicisotonic alternation. Also, loss of junctional integrity was visible in the LMO7 depleted NRK52E epithelial sheet under hypertonic-isotonic alternation.

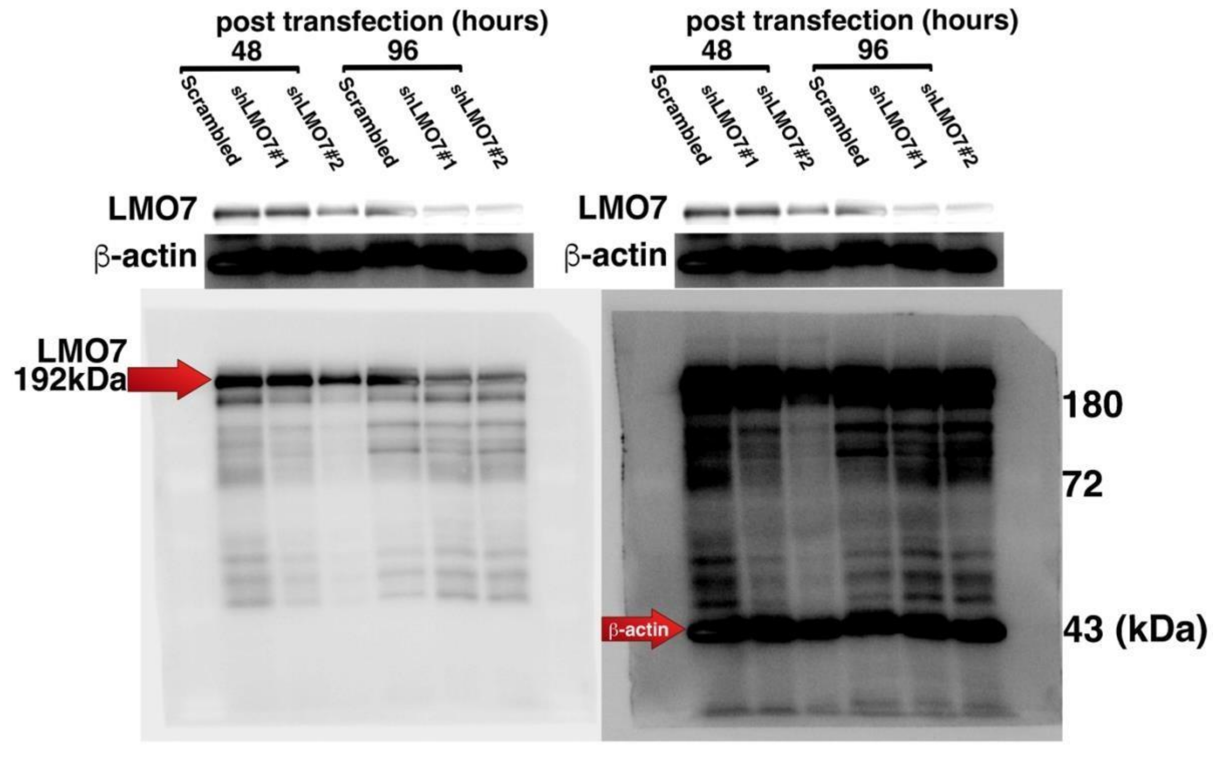

Figure S7. LMO7 depletion by small interfering RNA (shRNA) in NRK-52E cells**.** Western blot analysis indicated that the shLMO7#1 and shLMO7#2 transfection reduces endogenous LMO7 expression levels in NRK-52E cells.

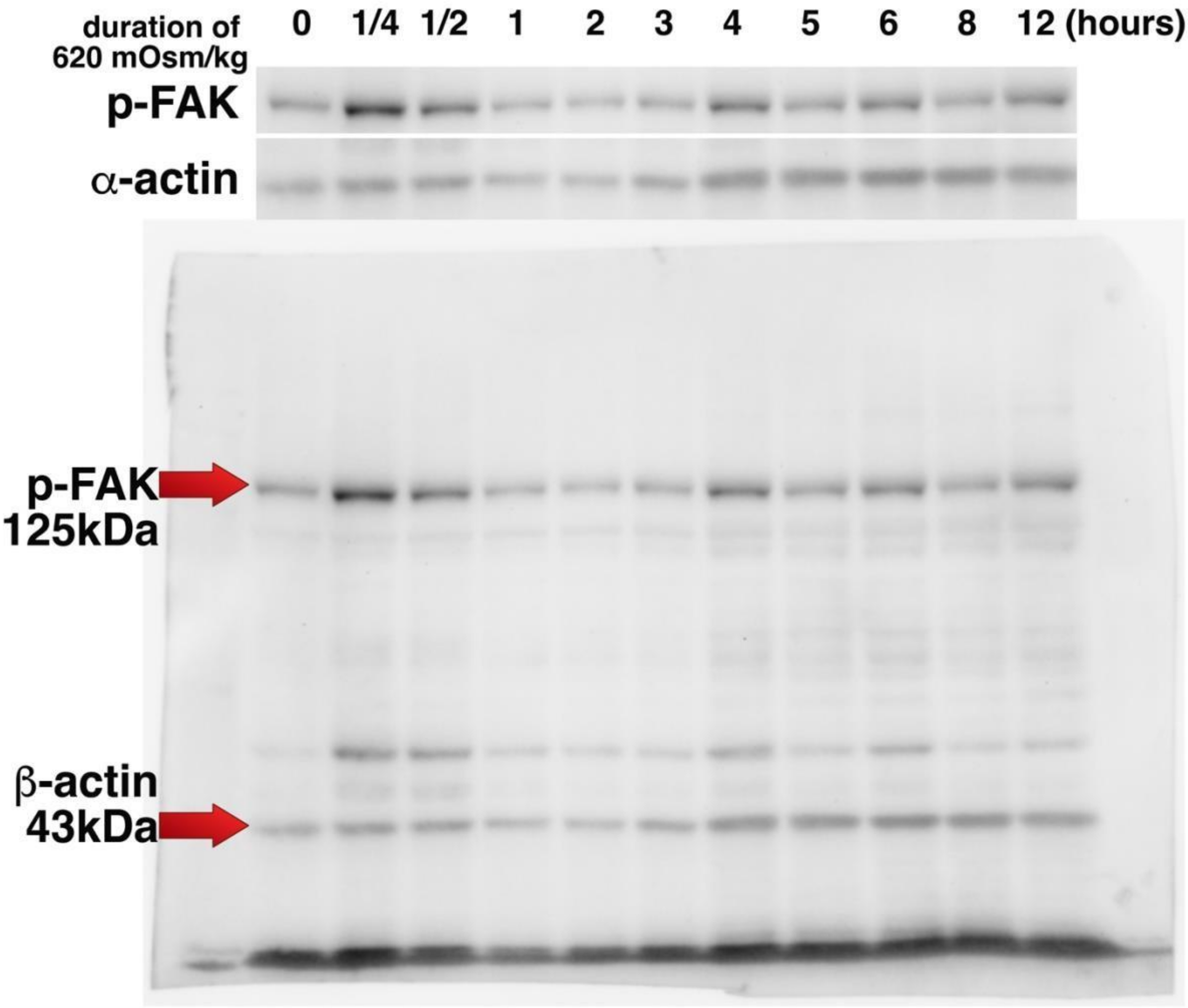

Figure S8. Western analysis examined FAK phosphorylated during 620 mOsm/kg administration.

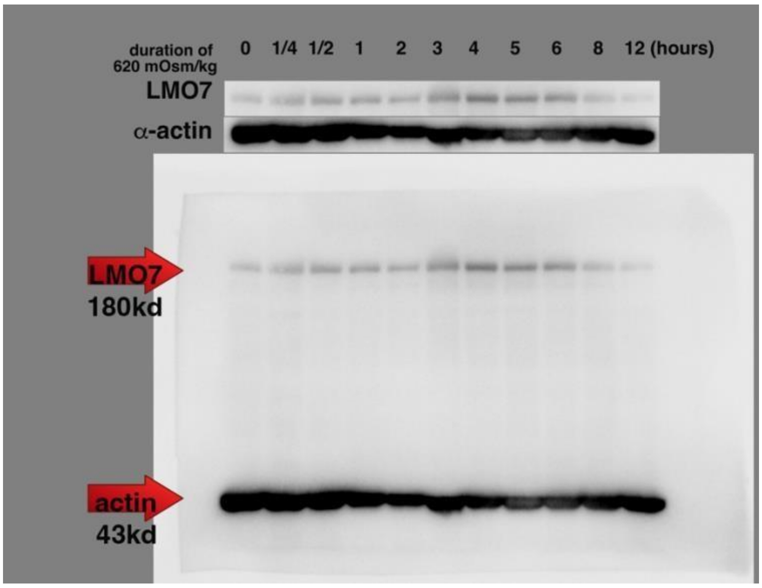

Figure S9. Elevation of LMO7 expression levels in NRK-52E cells by 620 mOsm/kg stimulation.

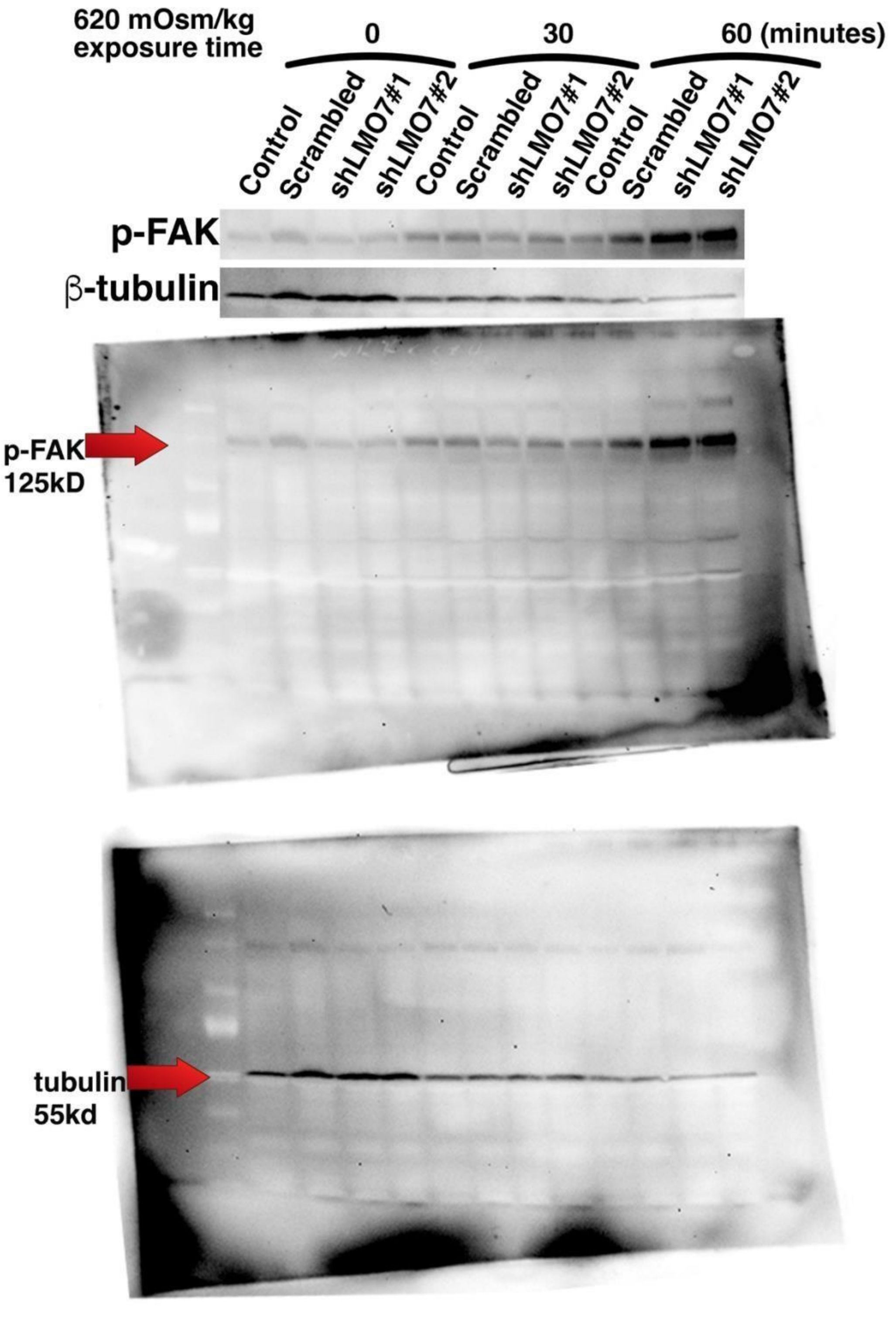

Figure S10. FAK phosphorylation in the LMO7 depleted NRK-52E cells is relatively higher than the NRK-52E cells without LMO7 depletion.

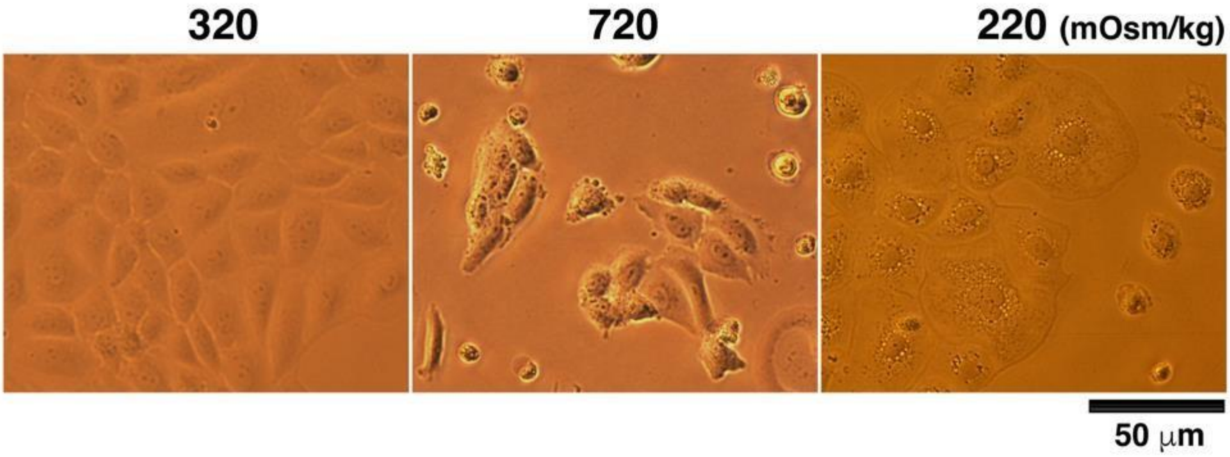

Figure S11. NRK-52E cells were damaged by osmotic stress. Under hypertonic or hypotonic condition, osmotic pressure forces push plasma membrane inward or outward. The osmotic pressure force pushes plasma outward cells got swelling when cells cultured in hypotonic medium. When cells cultured in hypertonic medium, the osmotic pressure force pushes plasma membrane inward and cells got shrinkage. The osmotic stress also caused cell death, as noted imaging, dead cells were observed in the hypertonic or hypertonic cultures .

Table S1. 241 proteins identified in 620 mOsm/kg conditioned medium.

Histone cluster 1 H1 family member c
Thioredoxin-like protein 1
AP-2 complex subunit beta
Protein phosphatase 2 (Formerly 2A), regulatory subunit A (PR 65), alpha isoform, isoform CRA_a
Eftud2 protein (Fragment)
Proliferation-associated protein 2G4
DEAH (Asp-Glu-Ala-His) box polypeptide 15 (Predicted), isoform CRA_b
EPS8-like 2
IQ motif containing GTPase activating protein 1 (Predicted), isoform CRA_b
Nuclear ubiquitous casein and cyclin-dependent kinase substrate 1
Far upstream element-binding protein 1
40S ribosomal protein S28
Bleomycin hydrolase
Heterogeneous nuclear ribonucleoprotein Q
AIP1, isoform CRA_a
T-complex protein 1 subunit epsilon
RNA binding motif protein, X-linked-like 1B
Protein SEC13 homolog
RCG31311
RCG50226, isoform CRA_a
Enhancer of rudimentary homolog
AHNAK nucleoprotein
Protein S100-A11
Transaldolase
Naca protein
Hnrnpl protein (Fragment)
Sf3b3 protein (Fragment)
Chaperonin containing Tcp1, subunit 6A (Zeta 1)
Hypoxanthine phosphoribosyltransferase (Fragment)

Leukotriene A-4 hydrolase
NSFL1 cofactor p47
Tropomyosin 1, alpha, isoform CRA_i
Microtubule-associated protein
Pls3 protein (Fragment)
Actin-related protein 2/3 complex subunit 4
Cleavage and polyadenylation specific factor 6, 68kDa (Predicted), isoform CRA_b
Protein-lysine 6-oxidase
Lupus La protein homolog
UDP-glucose:glycoprotein glucosyltransferase 1
Arp2/3 complex 34 kDa subunit
Proteasome activator complex subunit 2
Endoplasmic reticulum resident protein 29
Heterogeneous nuclear ribonucleoprotein A3
SPT16 homolog, facilitates chromatin-remodeling subunit
Proteasome subunit alpha type-6
Elongation factor 1-delta
Ribosomal protein (Fragment)
RAN GTPase-activating protein 1
Transcription intermediary factor 1-beta
Aly/REF export factor
RCG34610, isoform CRA_c
Proliferating cell nuclear antigen
Coatomer subunit beta
Alpha glucosidase 2 alpha neutral subunit (Predicted)
Acylphosphatase
Peptidylprolyl isomerase (Fragment)
Periostin
TRK-fused gene protein
Glia maturation factor beta
Polypyrimidine tract binding protein 1, isoform CRA_c
Heterogeneous nuclear ribonucleoprotein D0
Tubulin alpha chain
Phosphoserine aminotransferase
Cellular nucleic acid-binding protein
F-actin-capping protein subunit alpha-1
40S ribosomal protein S14
Thimet oligopeptidase
Sorbitol dehydrogenase
Eprs protein (Fragment)
SUMO-activating enzyme subunit 2
DNA helicase
Eukaryotic translation initiation factor 3 subunit A
Similar to ubiquitin-conjugating enzyme E2 variant 1
60S ribosomal protein L5
Eukaryotic translation initiation factor 2 subunit 1
Slit homolog 2 protein (Fragment)

Hsp90 co-chaperone Cdc37
Similar to tropomyosin 1, embryonic fibroblast-rat, isoform CRA_c
U5 small nuclear ribonucleoprotein 200 kDa helicase
S-phase kinase-associated protein 1
Peptidyl-prolyl cis-trans isomerase FKBP4
Na(+)/H(+) exchange regulatory cofactor NHE-RF1
Drebrin-like protein
Eukaryotic translation initiation factor 1
Vasodilator-stimulated phosphoprotein
DnaJ (Hsp40) homolog, subfamily B, member 4
Malate dehydrogenase, mitochondrial
Small nuclear ribonucleoprotein Sm D2
LIM domain 7
Splicing factor proline and glutamine rich
Tight junction protein 1 (Predicted)
Protein S100-A1
Retinal dehydrogenase 2
Staphylococcal nuclease domain-containing protein 1
Glycine--tRNA ligase (Fragment)
RAN-binding protein 1
Glyoxalase domain-containing protein 4
Coatomer subunit alpha
Calcyclin-binding protein
Septin-7
Platelet-activating factor acetylhydrolase IB subunit beta
Histone H1.5
Acidic leucine-rich nuclear phosphoprotein 32 family member B
40S ribosomal protein S12
T-complex protein 1 subunit eta
Non-POU domain-containing octamer-binding protein
Rab GDP dissociation inhibitor alpha
Protein kinase C substrate 80K-H
Stress-induced-phosphoprotein 1
Clathrin-assembly lymphoid myeloid leukemia protein
Plectin
U2 snRNP auxiliary factor large subunit
Arginine--tRNA ligase, cytoplasmic
Coatomer subunit gamma-1
Elongation factor 1-gamma
40S ribosomal protein SA
Hyou1 protein
LIM and SH3 domain protein 1
Dead end homolog 1 (Zebrafish)
Methanethiol oxidase
Septin 6 (Predicted), isoform CRA_b
60S acidic ribosomal protein P1
LIM domain and actin-binding protein 1

Caldesmon 1, isoform CRA_b
Protein O-glucosyltransferase 3
Serine/arginine-rich splicing factor 2
Prenylcysteine oxidase
Platelet-activating factor acetylhydrolase IB subunit alpha
40S ribosomal protein S3
Dynamin-1-like protein
Eukaryotic translation initiation factor 4A1
RuvB-like helicase
F-actin-capping protein subunit beta
Keratin, type I cytoskeletal 10
Dipeptidyl peptidase 3
Ube2l3 protein
Actin-related protein 2/3 complex subunit 1B
Caprin-1
Heterogeneous nuclear ribonucleoprotein F
Nuclear transport factor 2
Destrin
Cytoplasmic dynein 1 heavy chain 1
Far upstream element-binding protein 2
WD repeat-containing protein 1
Chromosome segregation 1-like
Protein transport protein SEC23
Radixin
Vascular endothelial growth factor C
KH domain-containing, RNA-binding, signal transduction-associated protein 1
Ptges3 protein
Lamina-associated polypeptide 2, isoform beta
Septin-2
BWK4
Actin, beta-like 2
F-actin-capping protein subunit alpha-2
Keratin, type II cytoskeletal 1
Xaa-Pro aminopeptidase 1
Latent-transforming growth factor beta-binding protein 3
Plasminogen activator inhibitor 1 RNA-binding protein
10 kDa heat shock protein, mitochondrial
Lamin-B1
Eukaryotic translation initiation factor 3 subunit B
Catenin (Cadherin associated protein), alpha 1
Biliverdin reductase B
Serine/threonine-protein phosphatase PP1-alpha catalytic subunit
Proteasome subunit alpha type-4
Sulfhydryl oxidase
Serine protease inhibitor
40S ribosomal protein S8
DNA-(apurinic or apyrimidinic site) lyase (Fragment)

N-acetylneuraminate synthase
Spectrin alpha chain, non-erythrocytic 1
Spliceosomal protein SAP155 (Fragment)
Nucleosome assembly protein 1-like 1
Protein arginine methyltransferase 1-like
Proteasome activator complex subunit 1
Galectin
Thioredoxin reductase 1, cytoplasmic
40S ribosomal protein S9
Carbonyl reductase [NADPH] 1
SUMO-conjugating enzyme UBC9-like
6-phosphogluconate dehydrogenase, decarboxylating
T-complex protein 1 subunit gamma
Heat shock 27kDa protein 1
Thymosin beta-10
FACT complex subunit SSRP1
RNA-binding protein EWS-like
Histone-binding protein RBBP7
Annexin A3
Exportin-1
Serine-threonine kinase receptor-associated protein-like
Importin subunit beta-1
C-C motif chemokine 20
Heterogeneous nuclear ribonucleoprotein C
Eukaryotic translation initiation factor 3 subunit E
Receptor of activated protein C kinase 1
Ribosomal protein S19-like
Nucleobindin 2, isoform CRA_b
Pre-mRNA-processing factor 19
Caveolae-associated protein 1
40S ribosomal protein S21
DEAD (Asp-Glu-Ala-Asp) box polypeptide 5 (Fragment)
Small nuclear ribonucleoprotein E
Glyceraldehyde-3-phosphate dehydrogenase
RCG45615, isoform CRA_a
Talin-1
Transgelin
Serpin H1
Coatomer subunit beta'
Peptidylprolyl isomerase
Actin-related protein 3
Nucleophosmin
Pyrophosphatase (inorganic) 1
Peroxiredoxin
Lactoylglutathione lyase
C-type mannose receptor 2
Regulator of chromosome condensation 2

FUS RNA-binding protein
MHC class I RT1.Aw3 protein
Prolylcarboxypeptidase
T-complex protein 1 subunit alpha
ELAV-like protein 1
Multifunctional protein ADE2
T-complex protein 1 subunit beta
Calmodulin-1
Adenylyl cyclase-associated protein 1
Clathrin heavy chain
RNA-binding protein 3
Osteopontin
Fatty acid synthase
Fibulin-5
Adenosylhomocysteinase
Inositol-1-monophosphatase
Histone H3
T-complex protein 1 subunit delta
Aspartyl aminopeptidase
Acidic leucine-rich nuclear phosphoprotein 32 family member E
RB-binding protein 4, chromatin-remodeling factor
Activated RNA polymerase II transcriptional coactivator p15 Poly(rC)-binding protein 3 von Willebrand factor A domain-containing protein 1

Table S2. Extracellular proteins in the secretome profiling of NRK-52E exposed to 620 mOsm/kg.

Gene Symbol	Protein Description	Function	Pathway	Remark
ACTR3	Actin-related protein 3	Actin nucleation	Actin dynamics	4
ALDH1	Aldehyde Dehydrogenase 1 Family Member A1	regulation of the metabolic responses to high-fat diet	Signaling by retinoic acid	4
ANP32B	Acidic leucine-rich nuclear phosphoprotein 32 family member B	Cysteine-type endopeptidase activity	Apoptotic pathway	4
ANXA3	Annexin A3	Inhibition of phospholipase A2	Prostaglandin regulation	4
ARPC1B	Actin-related protein 2/3 complex subunit 1B	Actin nucleation	Actin dynamics	4
ARPC2	Arp2/3 complex 34 kDa subunit	Actin nucleation	Actin dynamics	4
ARPC4	Actin Related Protein 2/3 Complex Subunit 4	Actin nucleation	Actin dynamics	4
BLMH	Bleomycin hydrolase	Cysteine peptidase	Unknown	4
BLVRB	Biliverdin reductase B	Heme metabolism	Metabolism of porphyrins	4
CACYBP	Calcyclin-binding protein	Calcium-dependent ubiquitination	Proteosomal degradation	4
CALM1	Calmodulin 1	Regulation on ion channel and aquaporins	RET signaling	4
CAPZA2	F-actin-capping protein subunit alpha-2	Barbed-end actin binding protein	Actin dynamics	4
CCL20	C-C Motif Chemokine Ligand 20	cytokine	inflammation	5

CCT6A	Chaperonin Containing TCP1 Subunit 6A	Component of TCP1 ring complex (TRIC)	Chaperonin-mediated protein folding	4
CLTC	Clathrin heavy chain	clathrin-coated vesicles	GAP junction trafficking	4
DNM1	Dynamin 1	GTP binding protein	Clathrin mediated endocytosis	4
DNPEP	Aspartyl aminopeptidase	Peptide metabolism	Unknown	4
DPP3	Dipeptidyl peptidase 3	Metallopeptidase	Cleaves and degrades bioactive peptides, such angiotensin, Leu-enkephalin, and Metenkephalin	4
DSTN	Destrin, an actin depolymerizing factor	Actin depolymerization	Actin Cytoskeleton remodeling	4
EPS8L2	EPS8-like 2	EPS8 gene family	Membrane ruffling and actin Remodeling	4
FBLN5	Fibulin 5	Extracellular matrix protein	Elastic fiber formation	5
FKBP4	FKBP prolyl isomerase 4	Cis-trans prolyl isomerase	Protein folding and trafficking	4
GLO1	Lactoylglutathione Lyase	Formation of S-latoylglutathione	TNF signaling	4
HSPB1	Heat shock protein family B member1	Molecular chaperone and protein folding	RET signaling	4
HYOU1	Hypoxia up-regulated 1	Protein folding and secretion	Unfolded protein response	4
LGALS1	galectin 1	β-galactoside-binding protiens	Modulating cell-cell and cell-ECM interaction	5
LOX	Protein-Lysine 6-Oxidase	crosslinking of collagen and elastin	Collagen and elastic fibre assembly	5
LTA4H	Leukotriene A4 Hydrolase	an enzyme contains both hydrolase and aminopeptidase activities	inflammation	4
LTBP3	Latent Transforming-growth factor β binding protein 3	Forming a complex with TGF-β	Apoptotic pathway	5
NACA	Nascent polypeptide associated Complex subunit α	Assembly of thin and thick filament	ERK signaling	4
NANS	N-Acetylneuraminate synthase	Generating phosphorylated forms of Neu5Ac	Biosynthetic pathway of sialic acids	4
PAFAH1B1	Platelet activating factor acetylhydrolase 1b	platelet activating acetylhydrolase	Organelle biogenesis	4
PAFAH1B2	α2 catalytic subunit of type I platlet-activating factor acetylhydrolase	platelet activating acetylhydrolase	Organelle biogenesis	4
PLEC	Plectin, actin-intermediated filament cross-linking factor	anchors intermediate filaments to desmosomes	Cell-junction organization	4
POSTN	periostin	secreted extracellular matrix protein	Oncogenic pathway	4
QSOX1	Sulfhydryl Oxidase 1	Catalyzing the oxidation of sulfhydryl groups in peptide thiols to disulfides	Cellular quiescence	5
RACK1	Receptor for activated C kinase 1	Scaffolding protein	TNF signaling	4
RDX	Radixin	Linking F-actin to plasma	Rho A pathway	4
S100A1	S100 calcium binding protein A1	member of the S100 family	Ca^{2+} homeostasis	4
S100A11	S100 calcium binding protein A11	member of the S100 family	Cell motility	4

SLC9A3	sodium/hydrogen exchanger regulatory cofactor	Na(+)/H(+) exchange regulatory cofactor NHE-RF1		4
SSP1	Secreted phosphoprotein 1, osteopontin	Cytokine, Cell-ECM interaction	Integrin pathway	5
TCP1A	A member of the chaperonin containing TCP1 complex (CCT)	Molecular chaperone complex, ATP dependent protein folding	Regulation on ciliogenesis, and transports vesicles to cilla	4
TXNRD1	Thioredoxin Reductase			
VASP	Vasodilator Stimulated Phosphoprotein	Cytoskeleton remodeling and cell polarity	Cell-junction organization	4
VEGFC	Vascular endothelial growth factor C	Angiogenesis, endothelial cell growth, Blood vessel permeability	VEGFR2 and VEGFR3 signaling	5
VWA1	Von Willebrand Factor A Domain Containing 1	extracellular matrix proteins		5

Confidence score for protein being released to outside of cell as an extracellular protein is evaluated with GeneCards version 5.9. The proteins that listed in secretome profiling are categorized to extracellular proteins, when extracellular localization was scored 4 and more.

Table S3. Detailed material information.

Ragent Or Resource	Source	Identifier	Working Status
**Antibodies**	**Company**		
Anti-LMO7	Santa Cruz		1/500 for Western and IF and 1/200 for IHC
Anti-p-FAK	cell signaling	Cat# 3281	1/500 for IF, 1/500 for Western
Anti-Emerin	Santa Cruz		1/500 for IF
Anti-β-catenin	Santa Cruz	Cat#	1/500 for IF
Anti-α-Actinin	Santa Cruz	Cat# SC-17829	1/500 for IF
Anti-p-Paxillin	Santa Cruz	Cat#SC-14035	1/500 for IF
Anti-Ecadherin	Arigo	Cat#ARG66195	1/1000 for IF
anti-ZO1	ThermoFisher	Cat#40-220	1/500 for IF
anti-NKCC2	cell signaling	Cat#	1/500 for IF, 1/200 for IHC
anti-β-actin	Arigo	Cat# ARG62346	1/10,000 for Western
Goat anti-mouse IgG-HRP	ROYEZ	Cat#C04001-2ML	1/10,000 for Western
Goat anti-Rabbit IgG-HRP	ROYEZ	Cat#C04003-2ML	1/10,000 for Western
Alex-488 donkey anti-mouse IgG	Jackson ImmunoRes	Cat#715545150	1/1,000 for IF
Alex-594 donkey anti-mouse IgG	Jackson ImmunoRes	Cat#715585150	1/1,000 for IF
Alex-488 donkey anti-rabbit IgG	Jackson ImmunoRes	Cat#711545152	1/1,000 for IF
Alex-594 donkey anti-rabbit IgG	Jackson ImmunoRes	Cat#711185152	1/1,000 for IF
**Chemicals, Enzymes and Materials**			
Immunohistochemical kit	BIOTnA	Cat#TAHC01D-100	
Hematoxylin	Leica	Cat#3801522	
DAPI	AAT BioQuest	Cat#17513	2μg/mL for IF
PF-573228	MCE	Cat#HY-10461	Stock: 10 μM
Phalloidin iFluro594	AAT BioQuest	Cat#23115	1/1,000 for IF
Urea	J.T. Baker	Cat#420301	Stock: 5M
NaCl	Sigma-Aldrich	Cat#7647-14-5	Stock: 5M
Phsophatase inhibiitor	Biotools	Cat#TAAR-BB13	
Protease inhibitor	Biotools	Cat#3Taar-BB12	
Ultrafiltration Vivaspin20	Sartorius	Cat#VCA002	
T-PER Tissue protein Ripa	ThermoFisher	Cat#78510	
Pierce BCA protein assay kit	ThermoFisher	Cat#23225	
PVDF membrane	Merck	Cat#IEVH00005	
Protein standard	Biomate	Cat#BR0671	
Protein standard	Biomate	Cat#BR1811	

chemiluminescence	ThermoFisher	Cat# 34096	
chemiluminescence	Visual Protein	Cat# LF08-500	
30% Acrylamide	Bionovas	Cat# AA0230-0500	
Triton X-100	Amresco	Cat#0694-1L	
Paraformylaldehyde	Merck	Cat#K28526995	
Fetal bovine Serum	Gibco	Cat#10437028	
Dulbeco;s Modified Eagle medium	Gibco	Cat#12100061	
Glutamine	ThermoFisher	Cat#25030081	
Penicillium/Stretomycin	Gibco	Cat#15140122	
Trypsin	ThermoFisher	Cat#25520056	
Lipofetamine 2000	ThermoFisher	Cat#11668030	
Opti-MEM	ThermoFisher	Cat#31-985-070	
Small interferring RNA, siLuciferase siLMO7#1 siLMO7#2			
Apoptosis detection kit	BioVision		Cat#K200
collagenase type II	Merk		Cat#9001-12-1
Hydrocortisone	Merk		Cat#H0888
Transferrin	Merk		Cat#T8158
Insulin	Merk		Cat#
**Others**			
Microscopy DM16000B	Leica		
Imaging system DFC360FC	Leica		
X-Citye XCT10A light source	Lumen Dynamic		
Panoramic MIDI digital scanner	3DHISTECH		
ChemiDoc System	Bio-Rad		
Flow cytometry	Backman		
Image-Pro Plus	MediaCybernetics		
Prism 8	GraphPad		
**Cell Lines**	**Company**	**Catalog Number**	**Medium**
NRK-52E	ATCC	ATCC CCL-1571	DMEM+10% FBS
